# Sex-specific behavioral outcomes of early-life adversity and emerging microglia-dependent mechanisms

**DOI:** 10.3389/fnbeh.2022.1013865

**Published:** 2022-10-04

**Authors:** Madison M. Garvin, Jessica L. Bolton

**Affiliations:** Neuroscience Institute, Georgia State University, Atlanta, GA, United States

**Keywords:** early-life adversity, sex differences, cognitive deficits, depression, substance abuse, microglia, synaptic pruning, CRH neurons

## Abstract

Early-life adversity (ELA) is known to alter brain circuit maturation as well as increase vulnerability to cognitive and emotional disorders. However, the importance of examining sex as a biological variable when researching the effects of ELA has not been considered until recently. This perspective discusses the sex-specific behavioral outcomes of ELA in both humans and animal models, then proposes microglia-mediated mechanisms as a potential underlying cause. Recent work in rodent models suggests that ELA provokes cognitive deficits, anhedonia, and alcohol abuse primarily in males, whereas females exhibit greater risk-taking and opioid addiction-related behaviors. In addition, emerging evidence identifies microglia as a key target of ELA. For example, we have recently shown that ELA inhibits microglial synapse engulfment and process dynamics in male mice, leading to an increase in excitatory synapse number onto corticotrophin-releasing hormone (CRH)-expressing neurons in the paraventricular nucleus of the hypothalamus (PVN) and aberrant stress responses later in life. However, ELA-induced synaptic rewiring of neural circuits differs in females during development, resulting in divergent behavioral outcomes. Thus, examining the role of microglia in the sex-specific mechanisms underlying ELA-induced neuropsychiatric disorders is an important topic for future research.

## Introduction

Early-life adversity (ELA) is now well-known to alter the development of multiple brain circuits and increase long-term risk for cognitive and emotional disorders, including depression and substance abuse (Gershon et al., 2013; Davis et al., 2017; Luby et al., 2017; Dahmen et al., 2018; Birnie and Baram, 2022; Hakamata et al., 2022). Decades of human research have established ELA, often defined as low socioeconomic status, poor family functioning, sexual or physical abuse, and/or an absence of parental nurturing, as a strong predictor of poor mental and physical health later in life (Springer et al., 2007; Vegt et al., 2009; Lovallo et al., 2013; Targum and Nemeroff, 2019). Rodent models of ELA have also been developed to provoke the cognitive and emotional dysfunction associated with mental disorders, so as to better elucidate their biological underpinnings (Baram et al., 2012; Molet et al., 2014; Rincón-Cortés and Sullivan, 2016).

Early on, studies of ELA identified aberrant reactivity of the hypothalamic–pituitary–adrenal (HPA) axis as a major outcome (Levine et al., 1991; Gilles et al., 1996; Nicolson, 2004; Tarullo and Gunnar, 2006). More recent research has allowed us to gain a better understanding of the mechanisms underlying abnormal stress reactivity, as well as other neural circuits involved. Several animal studies have found that ELA alters the development of brain regions involved in reward and stress, such as the nucleus accumbens, amygdala, and hypothalamus (Brenhouse and Andersen, 2011; Bolton et al., 2018a; Nieves et al., 2020; Wendel et al., 2021; Haikonen et al., 2022; Levis et al., 2022). In addition, recent work indicates that microglia, the resident immune cells of the brain, may play an important role in sculpting these developmental changes due to the critical role they play in synaptic pruning via engulfment of synaptic elements (Delpech et al., 2016; Hoeijmakers et al., 2017; Sellgren et al., 2017; Bolton et al., 2022). For example, we recently demonstrated that reduced synaptic engulfment by microglia during ELA leads to increased excitatory synapse number on corticotropin-releasing hormone (CRH)-expressing neurons of the hypothalamus and aberrant stress reactivity in adulthood (Bolton et al., 2022). However, few studies have investigated sex as a biological variable when examining the effects of ELA, even though sex differences are known to exist both in ELA’s later outcomes (Bath, 2020) and microglial development (Schwarz et al., 2012; Hanamsagar et al., 2017). In this perspective, we will highlight the sex-specific behavioral outcomes of ELA identified thus far and discuss emerging evidence for microglia-dependent mechanisms in an effort to catalyze this exciting area of research.

## Sex-Specific Behavioral Outcomes of Early-Life Adversity in Humans

Numerous studies have shown that ELA is associated with depression, substance abuse, and cognitive deficits in humans (reviewed in Dube et al., 2003; Short and Baram, 2019; Lemoult et al., 2020). For example, a study of institutionalized children found marked delays in cognitive development and decreased IQ, with the degree of impairment correlated with the duration of institutionalization (Nelson et al., 2007). Further studies have also shown that ELA may have sex-specific effects on cognition. In an assessment of ELA-induced cognitive dysfunction, an association between a history of childhood trauma and worse working and spatial memory in a standardized neuropsychological test battery was found in males only (Aas et al., 2011). In addition, ELA has been associated with a smaller hippocampal volume in men, which is a risk factor for cognitive deficits and other neuropsychiatric disorders. For example, Lawson et al. (2017) found that low socioeconomic status was associated with reduced hippocampal volumes in males but not females. Similarly, Samplin et al. (2013) revealed that childhood emotional abuse was associated with decreased hippocampal volume in males only. However, a history of emotional abuse was correlated with increased levels of depressive symptoms in both males and females (Samplin et al., 2013). These results indicate that while females might be more resilient to ELA-induced cognitive dysfunction and associated structural changes, they are not necessarily more resistant to its effects on emotional function.

ELA is a strong predictor of developing depressive disorders later in life (Mandelli et al., 2015). One of the most widely reported findings of individuals struggling with depression is hyperactivity of the HPA axis. Since ELA has also been found to increase the activity of the HPA axis, Heim et al. (2008) sought to determine if ELA was associated with an increased cortisol response to a CRH challenge in men with and without major depressive disorder (MDD). The study found that men with a history of childhood trauma showed an increase in ACTH and cortisol in response to a CRH injection as compared to controls. In addition, men with MDD that were exposed to childhood trauma exhibited increased responsiveness in comparison with controls and depressed men without childhood trauma (Heim et al., 2008). These results indicate that ELA leads to HPA axis hyperactivity in men, although women were not included in the study. Interestingly, other studies assessing ELA-induced mental illness found that women who were exposed to ELA were more likely to experience depression than men (Colman et al., 2013), suggesting that stress may manifest itself differently in men.

One way the long-term effects of ELA may manifest differently is through an increased susceptibility to drug and alcohol abuse, which has been observed in several studies on the sex-specific impact of ELA in humans. For example, Evans et al. (2017) found that the risk for substance abuse of a variety of different drugs was generally higher in men than in women exposed to childhood adversity. However, as the number of childhood adversity experiences increased, women’s risk for substance abuse disorders exceeded that of men (Evans et al., 2017). Furthermore, multiple studies examining the impact of ELA on alcohol abuse found that men had a higher risk of abusing alcohol than women (Strine et al., 2012; Colman et al., 2013). The higher rates of drug and alcohol abuse in men and the higher rate of depression diagnoses in women exposed to ELA suggest that while women may be more likely to seek treatment for psychiatric symptoms, men may be more likely to turn to drugs and alcohol as a coping strategy.

## Sex-Specific Behavioral Outcomes in Animal Models of Early-Life Adversity

In order to study the biological mechanisms of ELA, it is generally modeled in animals by manipulating either the quantity or quality of maternal care (Bolton et al., 2017). For rodents, two commonly applied models of ELA are maternal separation (MS) and limited bedding and nesting (LBN). Maternal separation is characterized by the daily separation of pups from the dam, ranging from one to eight hours a day over several days or even weeks (reviewed in Tractenberg et al., 2016). These repeated separations result in intermittent stress and an overall reduction in the quantity of maternal care (Pryce et al., 2005). The LBN model is applied continuously during an early sensitive period [typically postnatal days (P)2–9] by limiting the number of nesting materials provided and adding a mesh platform to the dam’s cage, which prevents the dam from constructing a typical, full nest. In this paradigm, the impoverished environment leads to fragmented and unpredictable maternal care, as measured by more frequent exits from the nest and shorter bouts of licking and grooming (Molet et al., 2016a; Walker et al., 2017). Although the majority of ELA models utilize rodents, non-human primate models have also been developed, including childhood maltreatment as a model of ELA for the rhesus macaque. In this paradigm, infants are placed with known abusive females who have been previously recorded engaging in infant abuse (McCormack et al., 2006). These animal models have all been shown to result in impairments to cognitive and emotional development, along with alterations in stress reactivity of the HPA axis (reviewed in Bolton et al., 2017; van Bodegom et al., 2017).

The LBN model has been widely used to investigate the impact of ELA on cognitive function, although historically, the sex-specific impact of ELA on cognitive development has not been well-defined. Recent studies have shown that ELA may have sex-selective effects on hippocampus-dependent memory deficits. For example, multiple studies have found that LBN provokes deficits in hippocampus-dependent spatial memory in a stress-free novel object location task in adult male (>P60) rodents (Molet et al., 2016b; Davis et al., 2017; Bolton et al., 2020; Xu et al., 2022). This cognitive dysfunction was also found in LBN male mice in a more stressful water-maze task (Naninck et al., 2015). The MS model elicited similar results, in which MS male mice showed impairments during spatial (Stoneham et al., 2021) and non-spatial memory tasks (Banqueri et al., 2017), as compared to controls. In contrast, in females, ELA did not result in hippocampus-dependent spatial memory deficits, as measured by the water maze (Naninck et al., 2015) and novel object location task (Loi et al., 2017). Interestingly, ELA did elicit memory impairments in the novel object location task transiently during adolescence (Bath et al., 2017). However, this deficiency did not carry into early adulthood, indicating that ELA impairment of spatial memory is more severe and long-lasting in males than females (Bath et al., 2017).

ELA has also been shown to impact emotional function, such as depressive-like behavior and reward-related behavior, in a sex-dependent manner in animal models. For example, we have found that ELA induces anhedonia, as measured by reduced sucrose preference and social play, in male rats (Bolton et al., 2018a). Similarly, recent work reported a decrease in cocaine- and opioid-seeking behavior in male ELA rats (Bolton et al., 2018b; Levis et al., 2022), suggesting that ELA induces anhedonia for both natural and drug rewards. In contrast, ELA in female rats provokes increased addictive-like behavior with opioids and no anhedonia (Levis et al., 2021). Interestingly, Okhuarobo et al. (2020) found that ELA escalated alcohol intake in male, but not female, mice, suggesting that the emotional dysfunction due to ELA may result in different types of “self-medicating” in males vs. females, in agreement with the human studies.

**Figure 1:**
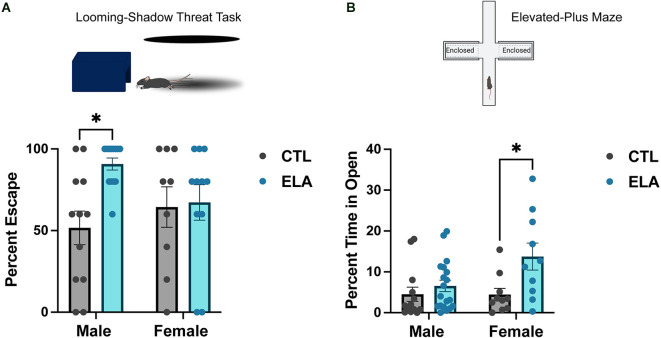
Sex-specific behavioral outcomes of early-life adversity. **(A)** ELA provokes an increased escape response to a looming-shadow threat in male, but not female, adult mice (trend for ELA x Sex interaction; *F*_(1,41)_ = 3.71, *p* = 0.06; *post-hoc* test, *p* < 0.05). **(B)** On the other hand, ELA increases risk-taking behavior, as measured by an increased percent time spent in the open arms in the elevated-plus maze, in female, but not male, adult mice (trend for ELA x Sex interaction; *F*_(1,48)_ = 3.31, *p* = 0.08; *post-hoc* test, *p* < 0.05). Figure created with https://biorender.com/. Data are mean ± SEM; *p* ≤ 0.1 criteria used for interaction terms to trigger subdivision for lower-order tests; ^*^p < 0.05 *post-hoc* test.

Because depression is linked with alterations of the HPA axis, it is not surprising that previous work has shown that ELA has a significant impact on HPA axis reactivity. For example, LBN rearing elevated plasma corticosterone by the end of the experience (P9), and this increase persisted into adulthood (Rice et al., 2008). Furthermore, these changes are accompanied by enhanced glutamatergic innervation and activity of CRH-expressing neurons in the PVN (Gunn et al., 2013; Bolton et al., 2022). Daviu et al. (2020) recently utilized the looming-shadow threat task as a behavioral read-out of PVN-CRH+ neuron activity. They discovered that an escape response to the simulated threat (i.e., looming shadow) is preceded by an increase in the activity of PVN-CRH+ neurons, and inhibiting their activity decreases the escape response (Daviu et al., 2020). When this behavioral test was applied in the context of ELA, it was revealed that adult LBN male mice escaped significantly more than control mice, in agreement with their increased PVN-CRH+ neuronal activity (Short et al., 2021; Figure 1A). In contrast, we have recently shown that LBN rearing from P2-P10 does not significantly alter the defensive behavioral response of adult female mice in the looming shadow task (Figure 1A), suggesting a sex-specific impact of ELA on the behavioral threat response in adulthood.

Some studies using MS as a model for ELA have shown increased anxiety-like behavior in MS mice, particularly in males (Daniels et al., 2004; Malcon et al., 2020; Tsotsokou et al., 2021), although this is inconsistent (Fabricius et al., 2008; Bondar et al., 2018; Wang D. et al., 2020). On the other hand, numerous studies applying the LBN model have found that exposure to ELA does not affect anxiety-like behavior in the open field or elevated-plus maze test in male rats and mice (Naninck et al., 2015; Molet et al., 2016a; Bolton et al., 2018a; Bolton et al., 2020, Figure 1B). Interestingly, we have recently found that female mice reared in LBN cages display increased risk-taking behavior in the elevated-plus maze test, spending more time in the open arms than controls (Figure 1B). Similarly, Viola and colleagues found that ELA in female mice decreased HPA axis reactivity, impaired risk assessment, and increased risking-taking in a task used to assess risk-taking under reward-seeking conditions (Viola et al., 2019). Together, these data suggest that ELA does not robustly impact anxiety-like behavior in males, but may even induce the opposite in females, by decreasing anxiety-like behavior and increasing risk-taking behavior.

## Emerging Microglia-Dependent Mechanisms

Microglia play an important role in regulating brain development, via pruning synapses and shaping neuronal function (reviewed in Delpech et al., 2015; Wu et al., 2015; Ngozi and Bolton, 2022). Notably, significant perturbations of microglial function during development due to injury, infection, or even stress can result in aberrant microglial synaptic pruning, thus causing the “rewiring” of brain circuits and ultimately behavioral changes. For example, deleting *Cx3cr1* in mice temporarily reduces microglial numbers during development, leading to deficient synaptic pruning, impaired functional brain connectivity, and deficits in social behavior (Zhan et al., 2014). As a result of findings such as these, the impact of environmental manipulations, such as ELA, on microglial function has become an important topic of research. Various human studies have identified inflammation as one potential mechanism by which ELA leads to poor behavioral outcomes later in adulthood (McQuaid et al., 2019; Pinto Pereira et al., 2019; O’Connor et al., 2020). However, stress can also inhibit and impair microglia. For example, Hoeijmakers et al. (2017) reported that LBN rearing altered microglial morphology and decreased Iba1+ staining coverage in the hippocampus of male mice at P9. Similarly, microglia in the hippocampus of P28 MS male mice exhibit increased expression of genes related to phagocytic activity and decreased expression of inflammatory genes relative to controls, indicating that ELA perturbs the function of microglia in the hippocampus (Delpech et al., 2016). In addition to the hippocampus, other studies have found that ELA disrupts microglial morphology and function in the prefrontal cortex (PFC) of male rodents during development (Reshetnikov et al., 2020; Wang R. et al., 2020), although females were not included in any of these experiments.

ELA may have differential effects on microglial function in males and females due to known sex differences in microglial maturation. In an analysis of microglia colonization in the developing brain, Schwarz et al. (2012) found that there are more microglia, and microglia of more “activated” (or less mature) morphologies, in certain regions, such as the hippocampus and amygdala, in males than in females early in postnatal life. Intriguingly, this balance shifts to the opposite pattern by puberty, such that adolescent and adult females have more “activated” microglia in the same brain regions (Schwarz et al., 2012). In the context of ELA, work by Bachiller et al. (2022) discovered that the extent of Iba1+ staining coverage is increased in the hippocampus of MS male, but not female, mice at P15. However, the same study found an increase in the percentage of microglia with an “activated” morphology in the PFC of MS females in comparison to male counterparts (Bachiller et al., 2022). Thus, it is possible that experiencing adversity during a developmental period in which microglia show sex differences in number and morphology in certain brain regions could impact males and females differently, thereby resulting in sex-specific and region-specific ELA-induced changes in synaptic pruning and ultimately divergent behavioral outcomes.

**Figure 2:**
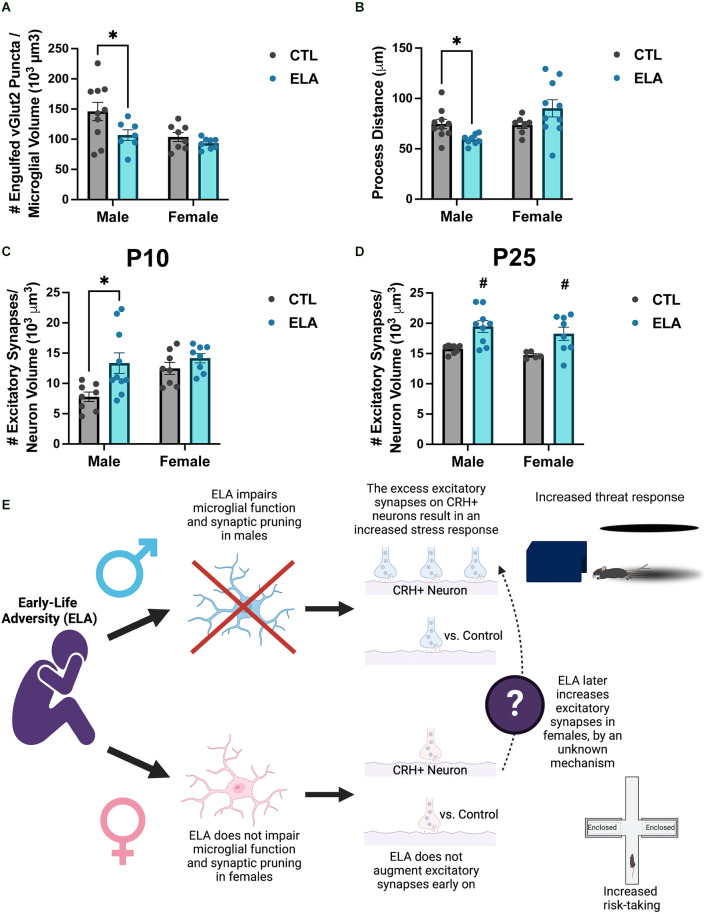
ELA alters microglial function and synaptic pruning in a sex-specific manner, leading to sex differences in the ELA-induced augmentation of excitatory synapse number on PVN-CRH+ neurons across development. **(A)** ELA diminishes the number of excitatory synaptic puncta engulfed by microglia at P8 in male, but not female, mice (trend for ELA x Sex interaction; *F*_(1,29)_ = 1.74, *p* = 0.1; *post-hoc* test, *p* < 0.05). **(B)** ELA inhibits the microglial process dynamics, as measured by total distance moved by microglial processes, at P8 in males, but not in females, even tending to do the opposite (significant ELA x Sex interaction; *F*_(1,29)_ = 7.38, *p* = 0.01; *post-hoc* test, *p* < 0.05). **(C)** ELA augments the number of excitatory synapses on PVN-CRH+ neurons at P10 in male, but not female, mice (trend for ELA x Sex interaction; *F*_(1,30)_ = 2.45, *p* = 0.1; *post-hoc* test, *p* < 0.05). **(D)** By P25, there is a significant increase in the number of excitatory synapses on PVN-CRH+ neurons in both male and female ELA mice (significant main effect of ELA; *F*_(1,26)_ = 17.5, *p* = 0.0003). **(E)** Conceptual figure displaying the proposed microglia-dependent mechanisms by which ELA results in sex-specific behavioral outcomes. Figure created with https://biorender.com/. Data are mean ± SEM; *p* ≤ 0.1 criteria used for interaction terms to trigger subdivision for lower-order tests; ^*^p < 0.05 *post-hoc* test; ^#^*p* < 0.05 main effect of ELA; adapted from Bolton et al. (2022).

Recent evidence shows that the ELA-induced disruption of microglial function described above may in fact cause the developmental circuit changes observed following ELA. We have previously shown that the ELA-induced anhedonia observed in male rats is accompanied by changes in the connectivity of reward- and stress-related circuits in the brain (Bolton et al., 2018a). Given the importance of microglia in shaping circuits, we then focused on the impact of ELA on microglia surrounding CRH-expressing neurons in the paraventricular nucleus (PVN) of the hypothalamus (Bolton et al., 2022). We found that ELA inhibited microglial function in P8 male CX3CR1-GFP+/– mice, both in terms of synapse engulfment (Figure 2A) and process dynamics (Figure 2B), leading to an increase in excitatory synapse number on PVN-CRH+ neurons at P10 and P25 (Figures 2C,D). Importantly, selective chemogenetic “reactivation” of microglia during ELA in males prevented the development of the synapse excess on PVN-CRH+ neurons and the aberrant stress response in adulthood (Bolton et al., 2022). However, female microglia were not significantly impaired by ELA at P8 (Figures 2A,B), and the developmental trajectory of the ELA-induced synaptic rewiring was different from males, although by P25 females also had increased numbers of excitatory synapses on their PVN-CRH+ neurons (Figure 2B). Together, these findings indicate that microglia play an important role in the sex-specific mechanisms underlying the ELA-induced behavioral outcomes described above (proposed mechanism illustrated in Figure 2E).

## Discussion

In this perspective, we present evidence that there are sex-specific behavioral outcomes of ELA in both humans and animal models, as well as microglia-dependent mechanisms that differ by sex. While techniques such as two-photon imaging are driving important discoveries in animal research, further advancements need to be made in technologies such as positron emission tomography (PET) to enable high-resolution functional imaging of microglia in humans. Importantly, many earlier studies excluded female subjects for simplification, due to the misconception that females presented more variability (Shansky, 2019). However, in recent years, researchers have begun to examine sex differences in ELA-induced cognitive deficits, affective disorders, and microglial dysfunction, thus making important contributions to our understanding of the outcomes of ELA and the underlying mechanisms. Moving forward, it is critical that we all include sex as a biological variable in our studies in order to fully capture the complexity of the enduring effects of ELA, as well as the promise for preventative interventions and treatments in all individuals.

## Data Availability Statement

The raw data supporting the conclusions of this article will be made available by the authors, without undue reservation.

## Ethics Statement

The animal study was reviewed and approved by the Institutional Animal Care and Use Committees of the University of California—Irvine and Georgia State University.

## Author Contributions

MG and JB wrote and edited the article, performed the experiments and analyzed the data. JB created the figures for the data included in this Perspective article. All authors contributed to the article and approved the submitted version.

## Funding

This work was supported by National Institutes of Health (NIH) grant K99/R00 MH120327 (JB).

## Conflict of Interest

The authors declare that the research was conducted in the absence of any commercial or financial relationships that could be construed as a potential conflict of interest.

## Publisher’s Note

All claims expressed in this article are solely those of the authors and do not necessarily represent those of their affiliated organizations, or those of the publisher, the editors and the reviewers. Any product that may be evaluated in this article, or claim that may be made by its manufacturer, is not guaranteed or endorsed by the publisher.
